# Intestinal permeability, digestive stability and oral bioavailability of dietary small RNAs

**DOI:** 10.1038/s41598-018-28207-1

**Published:** 2018-07-06

**Authors:** Jian Yang, Ismail Elbaz-Younes, Cecilia Primo, Danna Murungi, Kendal D. Hirschi

**Affiliations:** 1 USDA/ARS Children’s Nutrition Research Center, Baylor College of Medicine, 1100 Bates Avenue, Houston, TX 77030 USA; 2 Vegetable and Fruit Improvement Center, Texas A&M University, College Station, TX 77845 USA

## Abstract

Impactful dietary RNA delivery requires improving uptake and enhancing digestive stability. In mouse feeding regimes, we have demonstrated that a plant-based ribosomal RNA (rRNA), MIR2911, is more bioavailable than synthetic MIR2911 or canonical microRNAs (miRNAs). Here mutagenesis was used to discern if MIR2911 has a distinctive sequence that aids stability and uptake. Various mutations had modest impacts while one scrambled sequence displayed significantly enhanced digestive stability, serum stability, and bioavailability. To assess if small RNA (sRNA) bioavailability in mice could be improved by increasing gut permeability, various diets, genetic backgrounds and pharmacological methods were surveyed. An intraperitoneal injection of anti-CD3 antibody enhanced gut permeability which correlated with improved uptake of the digestively stable scrambled MIR2911 variant. However, the bioavailability of canonical miRNAs was not enhanced. Similarly, interleukin-10 (IL-10)–deficient mice and mice treated with aspirin displayed enhanced gut permeability that did not enhance uptake of most plant-based sRNAs. This work supports a model where dietary RNAs are vulnerable to digestion and altering gut permeability alone will not impact apparent bioavailability. We suggest that some dietary sRNA may be more digestively stable and methods to broadly increase sRNA uptake requires delivery vehicles to optimize gut and serum stability in the consumer.

## Introduction

Plants express an abundance of microRNAs (miRNAs) in their edible tissues^1^. Assuming plant miRNAs behave like the consumer’s endogenous miRNAs, where only partial sequence complementarity is needed to impact gene regulation, these miRNAs could be an efficacious solution to deliver disease suppressors or to fine-tune a consumer’s health^2^.

Several groups have reported that small RNAs (sRNAs) ingested from plant-based diets are bioavailable and act as bioactives^3–8^. The bioavailability and bioactivity of plant-based miRNAs in animals was first reported with a rice-based miRNA MIR168a, and some subsequent studies focused on the 26S ribosomal RNA (rRNA)-derived sRNA MIR2911^3,9^. MIR2911 is found in an assortment of vegetables and is bioavailable when mice are fed vegetable-enriched diets^9–12^. Unlike the dietary miRNAs we tested (i.e. MIR168), this sRNA is detectable in mouse sera and urine at levels proportional to the amount of sRNA present in the food^9^. Meanwhile, a significant amount of any synthetic form of a miRNA gavage fed to the animals causes only a slight increase in the detection of the molecule and is cleared rapidly when injected in the tail vein^11^. These findings suggest that plant-based modifications may aid in the bioavailability of specific sRNAs.

A principal concern is deducing the attributes that afford MIR2911 enhanced bioavailability. Studies suggest MIR2911 has three characteristics that appear to aid its bioavailability: first, a high GC content may increase digestive stability^3,10^; secondly, complexation with other compounds during or after uptake affords MIR2911 greater serum stability^10^; third, synthesis via 26S rRNA degradation increases MIR2911 levels in the gut. While MIR2911 is not a canonical miRNA, the question lingers as to how the primary sequence and structure of this sRNA impacts its digestive stability and bioavailability. Furthermore, do any insights gleaned from MIR2911 transfer to canonical dietary miRNAs like MIR168a?

Attempts to replicate the MIR168a work in its entirety have proven unsuccessful^4,13,14^. These experiments are technically difficult and some variation in experimental techniques occur between labs; however, these studies have all failed to measure dietary miRNA uptake in consumers utilizing a variety of dietary regimes^13,15,16^. These negative studies have merit as they establish that miRNA uptake is not ubiquitous^15,17,18^. These groups were measuring dietary miRNAs of limited abundance in the plant/food source tissue that are inherently unstable during digestion. A further shortcoming in these studies is the failure to realize that the nutrition status of consumers is in continual flux, and in addition that biological variability is great. Disease, hormonal levels, and nutrition imbalance are among the important factors that influence nutrient requirements^19^. None of these factors have been adequately investigated in terms of their impact on dietary nucleic acid absorption. Some evidence suggests that bioavailability of dietary sRNAs can be improved in pathological conditions. For example, the bioavailability of gavage fed synthetic sRNAs increased when mice were treated with cisplatin, a drug that induced an acute pathological condition^9^. Cisplatin alters both renal function and mucin metabolism while causing appetite suppression and weight loss^20^. Given these numerous deleterious effects, treating mice with cisplatin is not an ideal system to address how changes in gut permeability alter dietary RNA bioavailability. However, many genetic or pharmacological models for gut injury and increased permeability are available to address this question.

Here we are conducting basic science in a mouse model system to investigate the relationship between the primary sequence of MIR2911 and its digestive stability and bioavailability while also determining the impact of altering gut permeability on the bioavailability of synthetic and plant-derived sRNAs. Our results suggest that the broad-based efficacy of plant-based sRNAs requires implementing novel delivery strategies.

## Results

### MIR2911 digestive stability

**Figure 1:**
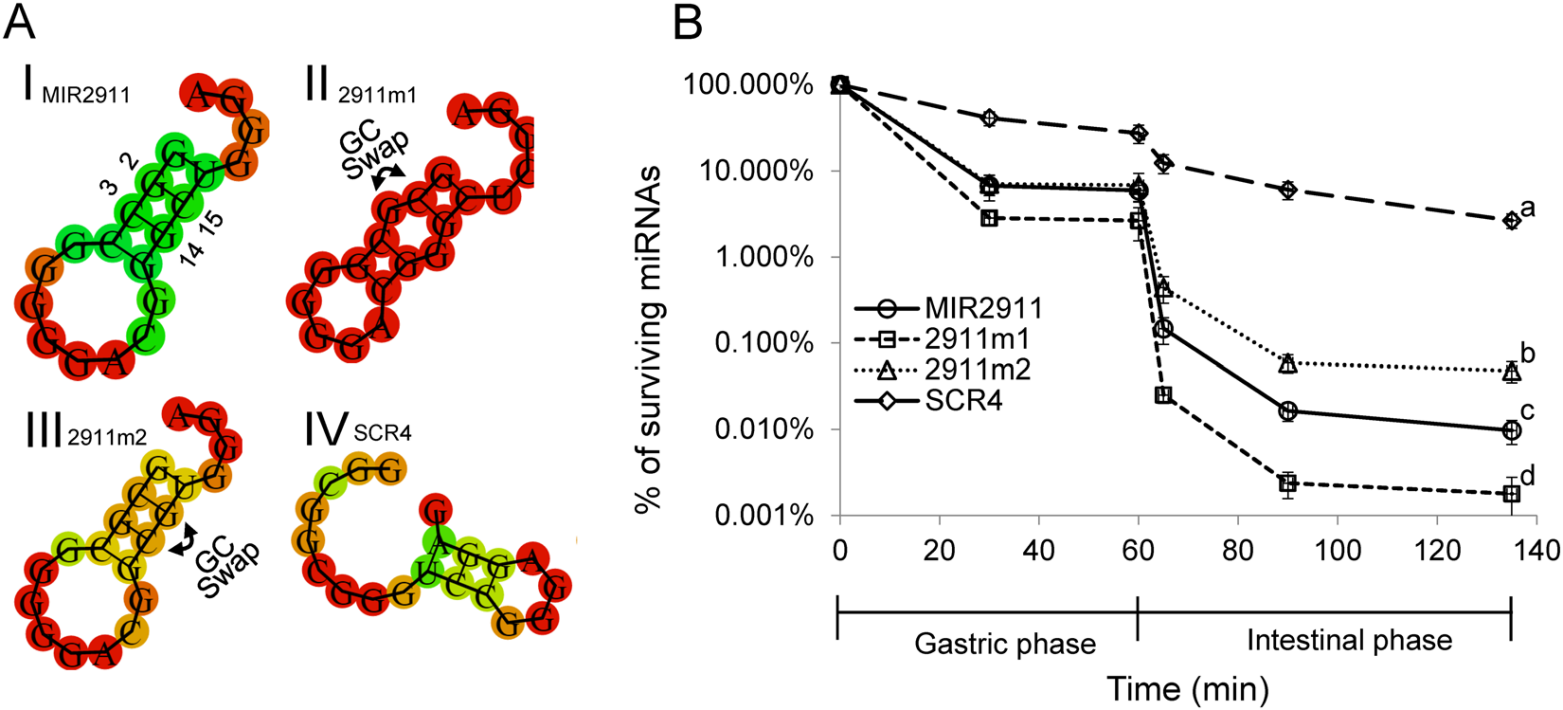
*In vitro* digestive stability of MIR2911 stem fold structure mutants. (**A**) RNA stem fold structures of MIR2911 and mutants. I. MIR2911; II. GC-swap mutant 2911m1: The G and C at the 2 and 3 position in MIR2911 is swapped to disrupt the stem structure; III. GC-swap mutant 2911m2: The G and C at the 14 and 15 position in 2911m1 is swapped so that the stem fold structure is reversed to that of original MIR2911. IV. scrambled-mutant SCR4: The entire MIR2911 sequence is scrambled, showing a different stem loop fold that is less thermodynamically stable. The free energy of the thermodynamic ensembles are: MIR2911 –6.56 kcal/mol; 2911m1 –6.00 kcal/mol; 2911m2 –5.67 kcal/mol; SCR4 –1.20 kcal/mol. RNA structure and free energy prediction were performed with RNAfold software. (**B**) Measurement of digestive stability of MIR2911, 2911m1, 2911m2 and SCR4 in the porcine *in vitro* digestion system mimicking gastric phase and intestinal phase. Cocktails of sRNAs were incubated in the *in vitro* digestion reaction buffers. Aliquots of reaction was sampled at 30 min and 60 min (0 to 60 min, gastric phase) and 65 min, 90 min and 135 min (60 min to 135 min, intestinal phase). The surviving percentage of each sRNA at each time point was calculated against the input level for each sRNA. n = 3. For the 135 min time point, mean values superscripted by the same letter are not significantly different at p ≤ 0.05.

Discerning the relationship between RNA primary structure and bioavailability may provide mechanistic insights into sRNA delivery. MIR2911 appears to be at least 10-fold more stable during *in vitro* digestion assays than traditional miRNAs^21^. Previously, work has inferred that MIR2911’s high GC content contributes to its digestive stability and bioavailability^3^. Here we synthesized variants of MIR2911 in order to characterize the relationship between primary sequence, putative structure and digestive stability of this sRNA. The first variant exchanged the G and C nucleotides at positions 2 and 3 (2911m1) to alter the putative stem-loop structure. Using 2911m1 the G and C at positions 14 and 15 were then exchanged to restore the original putative MIR2911 stem structure (2911m2). The third mutant (SCR4) scrambled the primary MIR2911 sequence in an effort to dramatically alter the structure and decrease the thermodynamic stability of the stem (the free energy of the thermodynamic ensemble is increased from about −6 kcal/mol for MIR2911 to −1.2 kcal/mol for SCR4) (Fig. 1A). All three variants maintained the GC content of the original MIR2911. To address digestive stability, an artificial *in vitro* digestion system that simulates mammalian gastric and intestinal conditions was then employed^10^. During *in vitro* digestion assays, the GC-exchange mutant (2911m1) had slightly decreased stability compared to MIR2911. Meanwhile, the 2911m2 mutant that attempted to restore the MIR2911 putative secondary structure was marginally more stable than MIR2911. The scrambled MIR2911 variant, while having the lowest thermodynamic stability, demonstrated the highest digestive stability-about 100-fold higher than that of MIR2911 (Fig. 1B).

**Figure 2:**
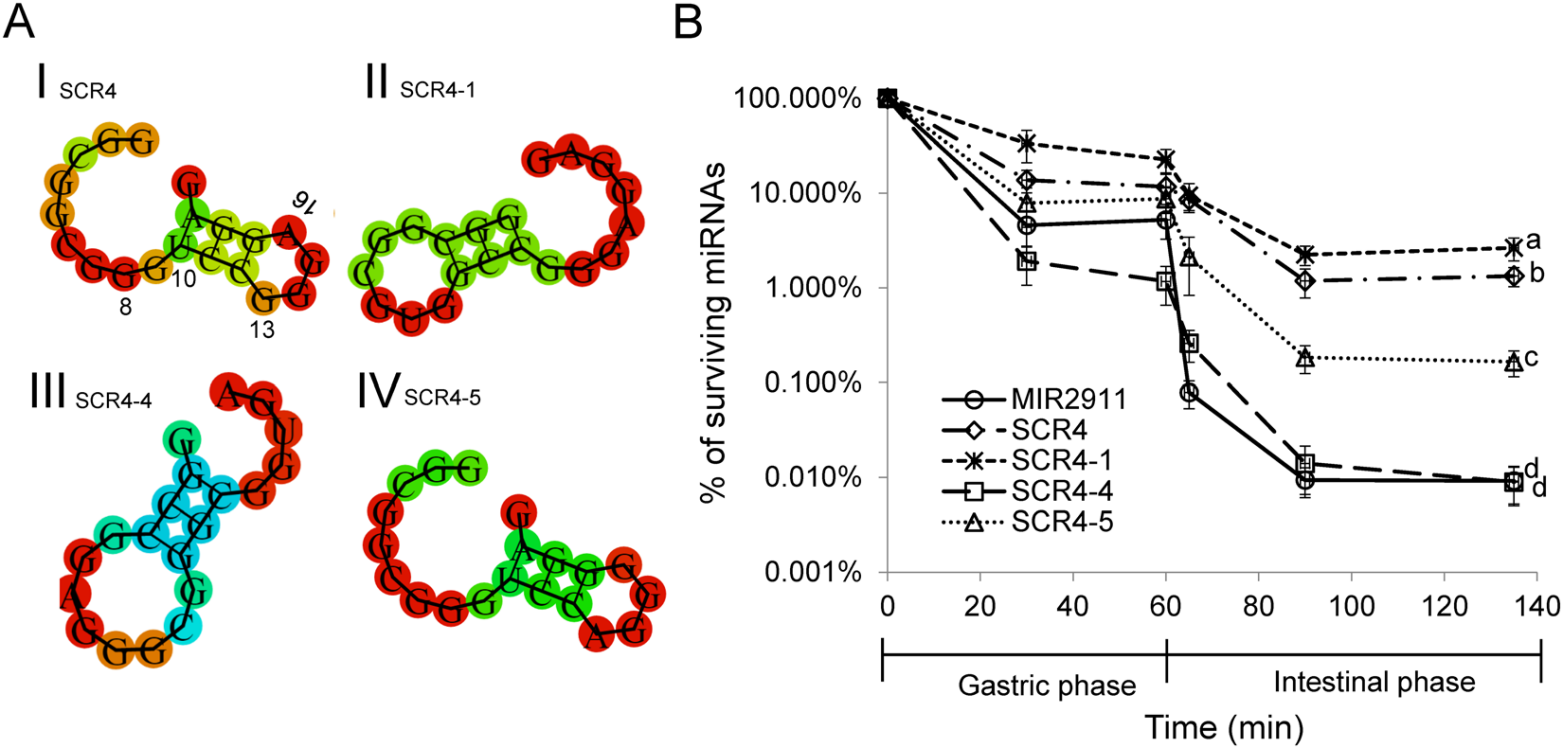
*In vitro* digestive stability of MIR2911, SCR4 and derivative mutants with modified “CA” or “CU” motifs. (**A**) RNA fold structures of SCR4 and mutants. I. SCR4; II. SCR4–1, removal of CU (by GU swap at positions 8 and 10 in SCR4) III. SCR4–4, removal of CA and CU (by GA swap at positions 7 and 10, and UG swap at positions 16 and 18 in MIR2911) IV. SCR4–5, creation of CA (by AG swap at positions 13 and 16 in SCR4–1). The free energy of the thermodynamic ensembles are: SCR4 –1.20 kcal/mol, SCR4–1 –5.71 kcal/mol, SCR4–4 –5.72 kcal/mol, SCR4–5 –2.37 kcal/mol. RNA structure and free energy prediction were performed with RNAfold software. (**B**) Measurement of digestive stability of MIR2911, 2911m1, 2911m2 and SCR4 in the porcine *in vitro* digestion system. Cocktails of sRNAs were incubated in the *in vitro* digestion reaction buffers. Aliquots of reaction was sampled at 30 min and 60 min (0 to 60 min, gastric phase) and 65 min, 90 min and 135 min (60 min to 135 min, intestinal phase). The surviving percentage of each sRNA at each time point was calculated against the input level for each sRNA. n = 3. For the 135 min time point, mean values superscripted by the same letter are not significantly different at p ≤ 0.05.

Given the stability of the scrambled variant SCR4, we sought to further characterize this sequence to probe the relationship between its primary sequence and digestive stability. The mammalian pancreatic Ribonuclease A (RNase A) is a major digestive enzyme for dietary RNAs and is known to have strong preferences in cleaving the bond between CU and CA motifs^22^. We attempted to alter the presence of CU or CA motifs by swapping nucleotides in SCR4 or MIR2911, while maintaining their GC content and overall nucleotide composition. In one variant, using SCR4 we removed the CU by swapping G and U at positions 8 and 10 (SCR4-1). In another, using MIR2911, we removed both the CA and the CU, by swapping G and A at positions 7 and 10, and swapping U and G at positions 16 and 18 (SCR4-4). In the third variant, using SCR4-1 we created a CA motif by swapping A and G at positions 13 and 16 (SCR4–5) (Fig. 2A). *In vitro*, the digestive stability of SCR4–4 was similar to MIR2911. The stability of SCR4–1 was marginally higher than that of SCR4 while the stability of SCR4–5 was greatly reduced compared to SCR4–1 (Fig. 2B).

### Bioavailability of MIR2911 variants

**Figure 3:**
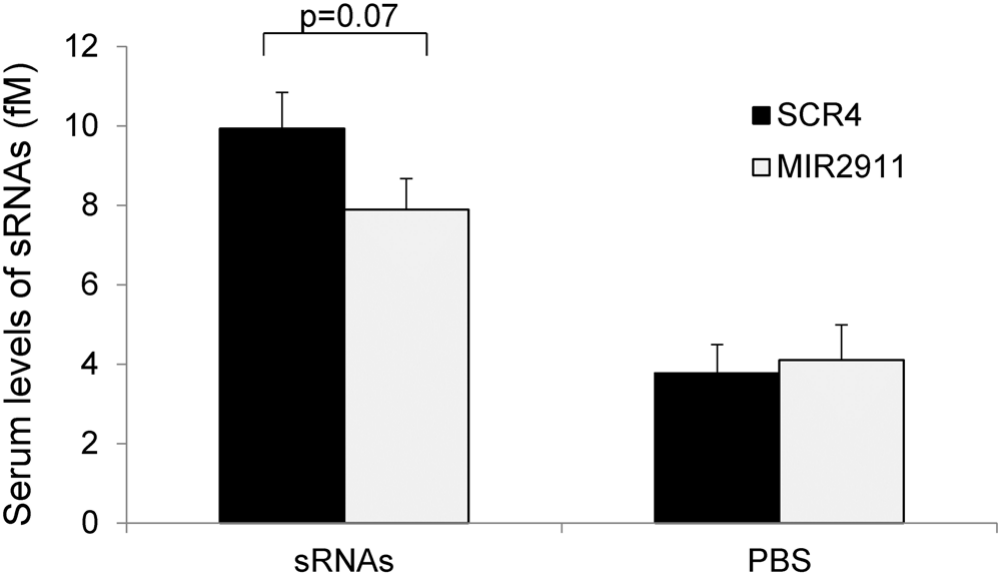
Bioavailability of gavage fed synthetic SCR4 and MIR2911 in mice. ICR mice were gavage fed either a single dose of sRNA mixture containing 400 pmols each of MIR2911 and SCR4 (sRNAs), or equal volume of PBS buffer (PBS). Serum was collected 1 h post-gavage feeding and analyzed by qRT-PCR for relative levels of SCR4 and MIR2911. Data was normalized to endogenous miR-16. n = 5.

**Figure 4:**
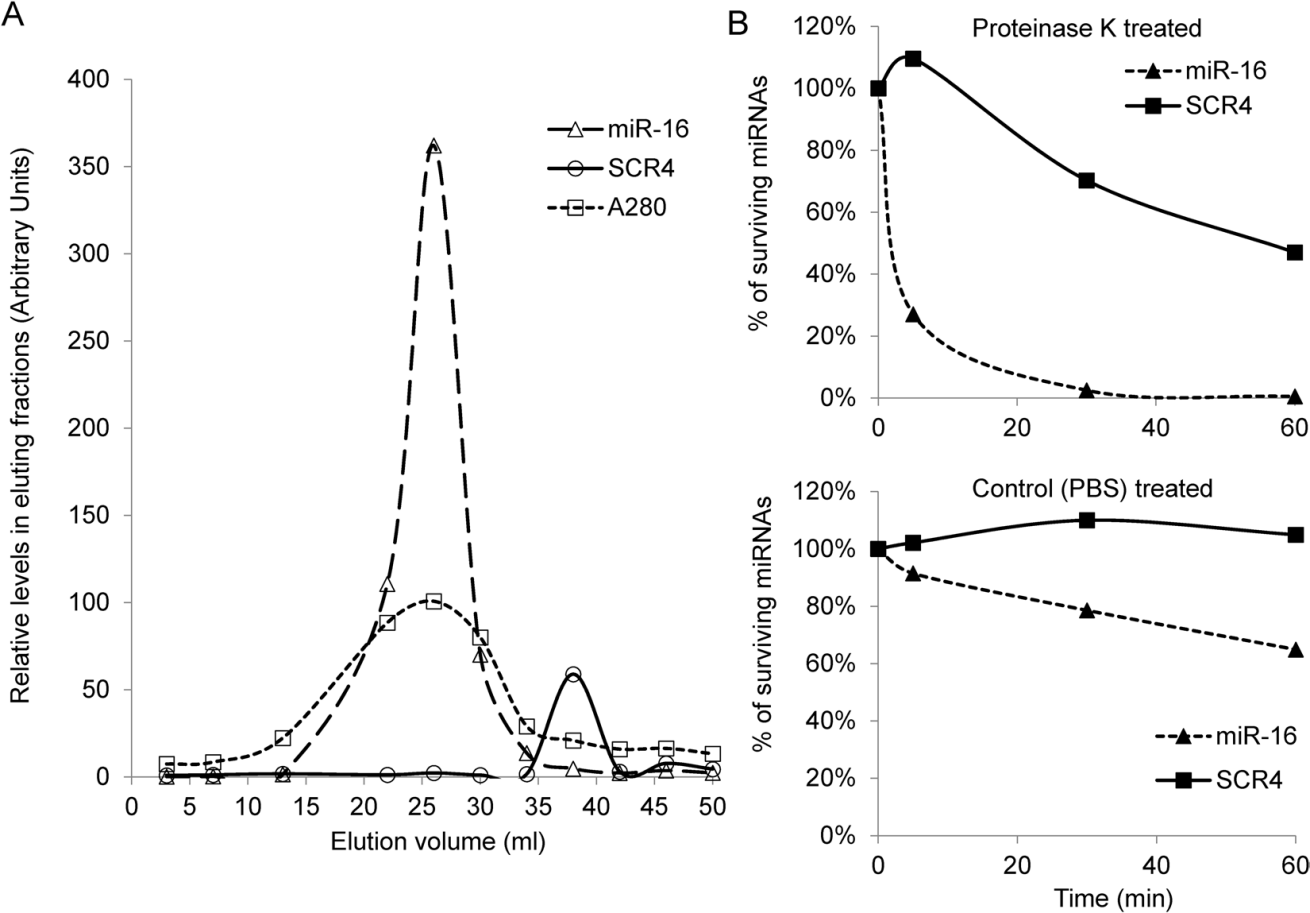
Circulating form of SCR4 in mice. (**A**) Size exclusion chromatography analysis of mouse sera containing SCR4. Mouse sera rich in SCR4 was loaded to a Sephacryl S-500 column and eluted with 50 mL PBS buffer. Relative levels of SCR4 and miR-16 in selected fractions were quantified by qRT-PCR. The protein abundance in each fraction was measured by spectral absorbance at 280 nm. (**B**) Measurement of resistance of serum SCR4 to proteinase K treatment. Mouse sera rich in SCR4 were mixed with either proteinase K (top panel) or PBS buffer (bottom panel). Aliquots of the reactions were sampled at 5 min, 30 min and 60 min to measure surviving levels of SCR4 or miR-16 by qRT-PCR. For both panel (A,B), a representative experiment from three biological repeats was shown.

Stability of SCR4 was greater than that of the synthetic MIR2911 (Figs 1 and 2); however, does this translate into improved bioavailability? To test this, we sought to compare and contrast uptake efficiency of MIR2911 and SCR4 by gavage feeding. Our previous work, along with others, demonstrated the inefficiency of uptake of synthetic RNAs, thus high dosages of the synthetic RNAs are required to obtain measurable serum levels (400 pmoles single dose per mouse)^9,11^. Using these conditions, an approximate 25% increase in the serum SCR4 levels was obtained compared to MIR2911 (Fig. 3). This suggested that SCR4’s enhanced digestive stability partially translated into enhanced bioavailability. In previous work, we used size exclusion chromatography (SEC) to characterize the circulatory forms of MIR2911^10^. Exosome-associated miRNAs (such as Let-7a) elute from SEC columns early relative to protein-associated miRNAs (such as miR-16)^23^. Copies of serum-derived SCR4, miR-16 were quantified in SEC fractions by qRT-PCR. The fractionation of miR-16 was consistent with previous work as it co-migrated with proteins (shown by A280), while SCR4 fractionated after the protein-associated miR-16 (Fig. 4A). To discern whether SCR4 is protein bound, proteinase K digestion assay was performed on sera enriched of SCR4. Agreeing to previous results, the Ago2-bound miR-16 was sensitive to proteinase K digestion, while SCR4 was largely resistant to the treatment (Fig. 4B).

### Serum Stability of MIR2911 Variants

**Figure 5:**
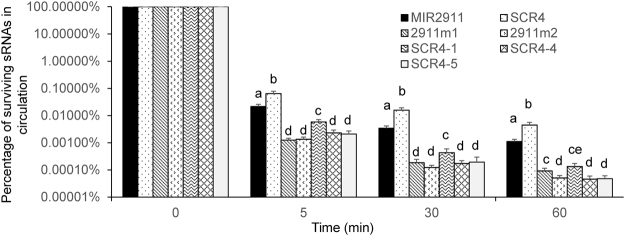
Serum stability of synthetic MIR2911, SCR4 and their mutant variants via tail vein injection. Cocktails of sRNAs containing MIR2911, SCR4 and other variants were injected into the mice through the tail vein. Blood was collected from mice at 5 min, 30 min and 60 min post injection. The levels at time 0 min were measured by adding sRNAs directly into Trizol/serum mixture and graphically this was used as a calibrator of 100% for subsequent time points. The percentage of surviving sRNAs in circulation at time points was calculated against the levels at time 0. n = 3. Mean values within a column superscripted by the same letter are not significantly different at p ≤ 0.05.

After uptake from the gut, circulating sRNAs face a gauntlet of challenges including limited stability in the blood and rapid renal clearance^24^. Could the variants of MIR2911 generated here be more serum stable? To test, we assayed the time course for survival of these synthetic sRNAs injected into the mouse tail vein. As many previous reports have documented^25^, clearance of these sRNAs was rapid. However, SCR4 and MIR2911 displayed the slowest clearance compared to other variants (Fig. 5).

### Mouse models to increase intestinal permeability

The mutagenesis studies with MIR2911 provided a more robust synthetic sRNA, SCR4, to use in gavage feeding studies to probe the relationship between intestinal permeability and bioavailability. Intestinal permeability controls material passing through the gastrointestinal tract. A healthy intestine has some permeability; however, increased permeability may contribute to enhanced uptake of dietary sRNAs. Previously established inflammatory bowel disease (IBD) mouse models may provide models to investigate dietary RNA uptake. Dextran sulfate sodium (DSS)-induced colitis in mice is widely used because of its simplicity and similarities to human ulcerative colitis^26^. However, our preliminary studies suggest this treatment causes DSS accumulation in the sera inhibiting RT-PCR reactions (data not shown). Thus, this popular model of IBD cannot be easily used to investigate dietary RNA uptake and bioavailability.

**Figure 6:**
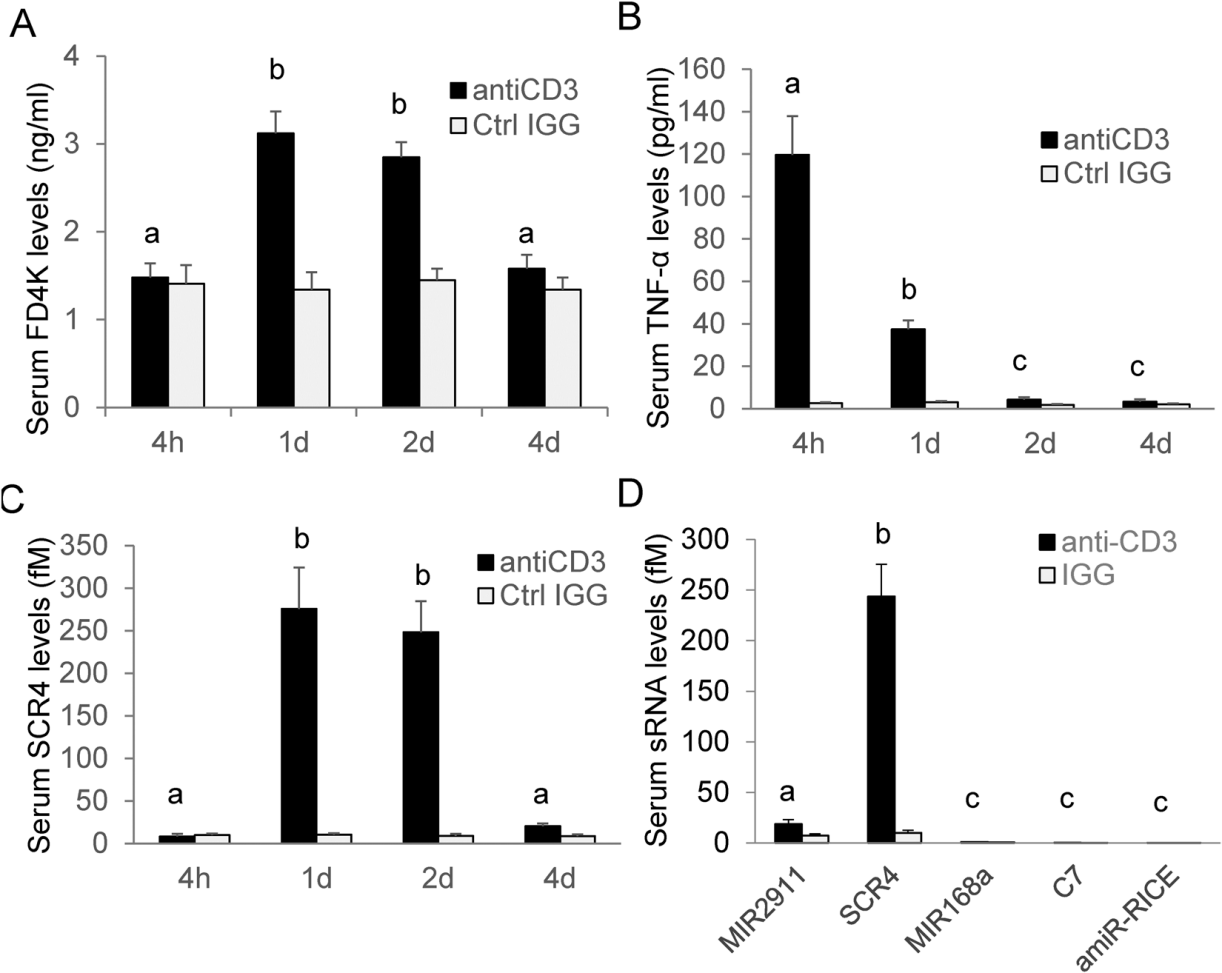
Uptake of dietary synthetic small RNAs in anti-CD3 antibody-induced gut injury mouse model. In panels (**A–C**), ICR mice were either treated with anti-CD3 antibody (anti-CD3) or the hamster IgG isotype control (Ctrl IGG) for 4 h, 1d, 2d and 4d. In panel D) the treatment was 1d. (**A**) Gut permeability assayed by determining the serum concentration of FITC-dextran 4000 (FD4K) gavage fed to mice 4 h after gavage feeding of FD4K. (**B**) TNF-α levels in serum. (**C**) Bioavailability of gavage fed single dose of 400 pmole synthetic SCR4 as determined by qRT-PCR of serum SCR4 1 h after feeding. (**D**) Comparison of uptake efficiency of various small RNAs. A cocktail of sRNAs including MIR2911, SCR4, MIR168a, C7 and amiR-RICE at 400 pmoles each were gavage fed to mice treated with anti-CD3 antibody or IgG control for 1d. Serum levels of the sRNAs were analyzed 1 h after feeding. n = 4. Mean values within a column superscripted by the same letter are not significantly different at p ≤ 0.05.

In previous work an intraperitoneal injection of anti-CD3 monoclonal antibody (Clone 145–2C11) into mice induced mucosal flattening and rapid, bi-phasic intestinal epithelial cell (IEC) apoptosis^27^. In the first, early phase, villous apoptosis was observed up to 4 h after injection, and in the second, later phase, apoptotic crypt cells gradually accumulated for up to 24 h. Fluorescent-labeled dextrans offer a simple tool for the evaluation of semipermeable membrane function. The anti-CD3 antibody-treated mice showed higher levels of FITC-dextran 4000 (FD4K) in sera than controls treated with IgG (hamster IgG isotype control) suggesting that this treatment altered gut permeability 24–48 h (Fig. 6A) without causing significant damage to other tissues (Supplementary information Fig. S1). Additionally, previous reports suggest that anti-CD3 antibody induces a polyclonal T cell activation resulting in the release of several cytokines, including tumor necrosis factor-alpha (TNF-alpha)^28^. In our anti-CD3 antibody treatments, we confirmed the increased levels of TNF-alpha post treatment and that mice maintained a normal growth for weeks after treatment (Fig. 6B and data not shown).

**Figure 7:**
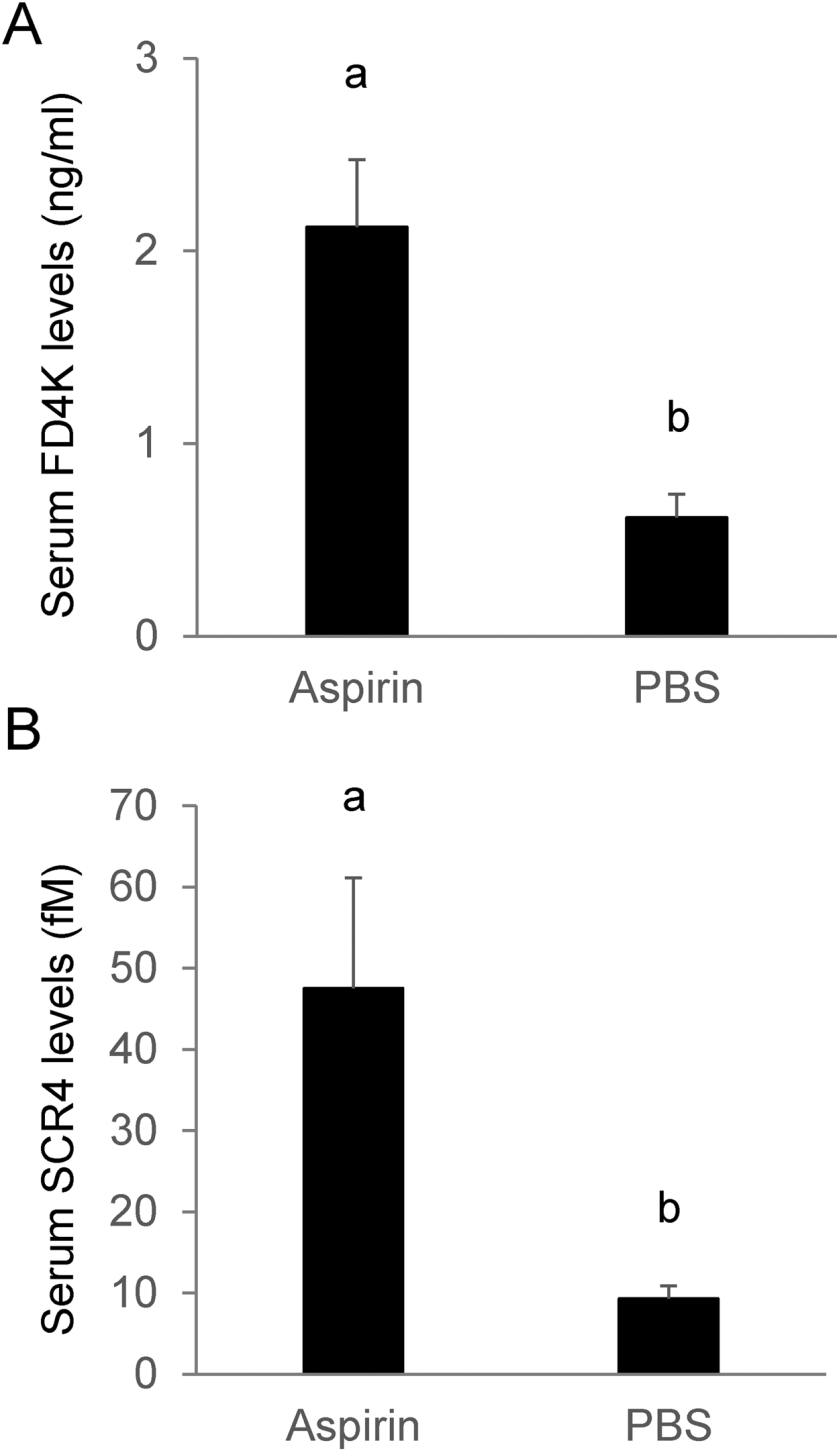
Uptake of SCR4 in aspirin-induced gut injury model. ICR mice were treated with oral dose of 200 mg/kg body weight aspirin (Aspirin) or PBS buffer (PBS) daily for 5 days before they were gavage fed FITC dextran-4000 FD4k and a single dose of 400 pmole of SCR4. (**A**) Gut permeability assayed by determining the concentration of FITC-dextran 4000 FD4K in the serum 4 h after gavage feeding of FD4K. (**B**) Serum SCR4 levels in mice was determined by qRT-PCR 1 h after gavage feeding of the sRNA. n = 5.

Chronic daily low dose and acute high dose aspirin treatment are also widely used models to study gut injury and perturbation in gastrointestinal permeability^29^. A high dose of 200 mg/kg aspirin^30^ was administered once daily by gavage for 5 days to mice to induce acute gut injury. In contrast to corresponding saline-treated mice, the majority of aspirin-treated mice displayed increased gut permeability (Fig. 7A).

We also used mouse mutants to assess the role of gut permeability and dietary sRNA uptake. IL-10-deficient mice have immune system dysregulation and have been used as a model for Crohn’s disease^31^. Two-week-old IL-10-deficient mice show an increase in gut permeability in the absence of any histological injury^32^. These mice appeared to be phenotypically indistinguishable from wild-type mice at 10-weeks of age; however, they develop colitis after approximately 12 weeks (data not shown).

### Intestinal permeability correlates with the bioavailability of SCR4

The temporal changes in gut permeability caused by anti-CD3 antibody treatment are associated with changes in bioavailability of gavage fed SCR4 (Fig. 6C). For example, four hours after anti-CD3 antibody injection there was no change in permeability or bioavailability; however, one day to two days after treatment both permeability and bioavailability increased. After four days, permeability and SCR4 bioavailability returned to levels seen in control mice. This relationship between permeability and bioavailability was also seen in mice treated with aspirin (Fig. 7B). When the IL-10-deficient mice were treated with anti-CD3 antibody there were no synergistic effects on uptake of SCR4 (Supplementary information Fig. S2).

**Figure 8:**
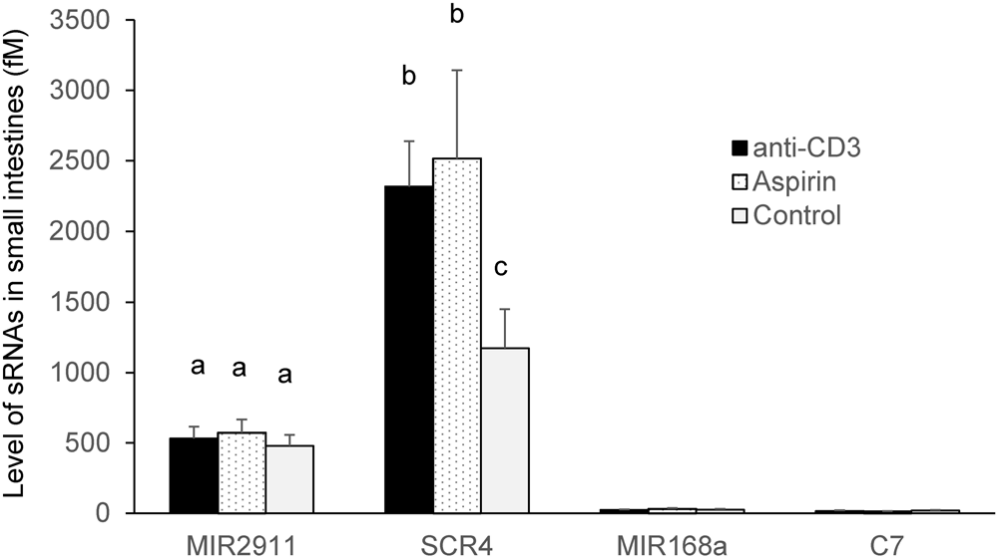
*In vivo* digestive stability of synthetic sRNAs in the small intestines of mice treated with anti-CD3, or aspirin. Mice were treated with anti-CD3 antibody (anti-CD3) or aspirin (Aspirin) for 5 days. In control group (Control), mice were intraperitoneally injected with hamster IgG isotype control. All mice were gavage fed a cocktail of synthetic sRNAs including 400 pmoles each MIR2911, SCR4, MIR168a and C7. The content of small intestines were analyzed for the surviving levels of the sRNAs 1 h after feeding. The concentration was calculated based on the assumption of 1 mL small intestine liquid volume. n = 5. Mean values within a column superscripted by the same letter are not significantly different at p ≤ 0.05.

Strikingly, SCR4’s bioavailability is far more enhanced in the anti-CD3 antibody-treated mice (over 20-fold increase over control), compared to MIR2911 (about 2-fold increase over control) (Fig. 6D). Although uptake of SCR4 was enhanced when permeability was heightened, the uptake of canonical miRNAs tested (i.e. MIR168a, C7 and amiR-RICE) was not significantly improved (Fig. 6D). Additionally, the anti-CD3 antibody treatment did not enhance the bioavailability of MIR2911 when mice were fed vegetable-rich diets (data not shown). We then measured the digestive stability of the sRNAs in the small intestine of animals treated with aspirin and anti-CD3 antibody (Fig. 8). Although the digestive stability of MIR2911 and a handful of miRNAs was not altered by the two treatments, the anti-CD3 and aspirin treatment appeared to slightly stabilize SCR4 within the intestine.

## Discussion

This study addresses a pressing set of questions pertaining to the bioavailability of dietary sRNAs. Multiple studies, including our own, have discounted the initial description of bioavailability of plant-based miRNAs^7^. Meanwhile, other groups suggest that dietary milk-based miRNAs are bioavailable because they are protected by exosomes^33–35^. We have detected a plant-based dietary sRNA (20 nt long) termed MIR2911 in consumers. Our previous work demonstrates that MIR2911 increases in abundance post-harvest in parallel with a unique circulatory packaging characteristic that aids its bioavailability^21^. Here our *in vitro* digestion studies of various MIR2911 mutants demonstrate that the primary sequence, structure, or presence of certain RNases digestion motifs can potentially contribute to bioavailability by impacting digestive stability. Our work demonstrates the scrambled MIR2911 variant SCR4 is more stable than MIR2911 without supplying a mechanistic understanding of this stability. Our findings do demonstrate that the GC content is not the sole determinant of this digestive stability, as all the MIR2911 variants tested had identical GC content and nucleotide composition.

Compared to canonical miRNAs, MIR2911 is more stable in digestion assays and during boiling^3^. Synthetic MIR2911 and the majority of MIR2911 mutants created here were rapidly degraded during *in vitro* digestion (Figs 1 and 2). This confirms work done by various groups demonstrating that the bulk of dietary sRNAs, including miRNAs, are almost completely digested when reaching the small intestine^36^. In agreement with our previous observations, we found that MIR2911 was more stable than the handful of synthetic miRNAs administered by tail vein injection^11^. Previous experiments show that lowering the GC content appears to decrease the digestive half-life of this RNA^3^. However, the present study suggests that beyond GC content, changes in sequence, structure, and putative RNase A substrate motifs can impact digestive and/or serum stability (Figs 1, 2 and 5). Due to the low overall digestive and serum stability, gavage-fed synthetic miRNAs and most sRNAs are not likely to be available for systemic absorption (Figs 3 and 6D).

The MIR2911 analysis provides a step forward in the understanding sRNA digestive stability. The scrambled MIR2911 (SCR 4) was more stable in both the digestion assay and in the tail injection studies (Figs 1, 2 and 5). The digestive stability of SCR4 was associated with improved bioavailability (Fig. 3); however, the large difference in digestive stability equated to only a modest improvement in bioavailability in healthy mice (Fig. 3). Our working hypothesis is that dietary RNAs will need to be exceedingly stable during digestion to see an expansive change in bioavailability. Our analysis of the circulating form of SCR4 (Fig. 4) suggests that this sRNA is not bound to a protein or encapsulated in an exosome. It is interesting to compare this variant of MIR2911 to RNA aptamers, short oligonucleotides forming a stable 3D structure with other molecules. One attractive hypothesis is that this RNA variant binds to certain circulatory proteins or other types of macromolecules of similar size once entering the bloodstream. This protease-resistant binding partner could potentially be identified by using affinity pull-down approaches. Viroid ssRNAs (single-stranded RNAs) encode no proteins and are stable without a protective protein capsid or coat^37–39^. Plants may produce a small handful of these types of ultra-stable sRNAs (not miRNAs) that may be bioactive. In the future, it might also be interesting to test, when expressed exogenously in plants, whether SCR4 would have similar complexation to that of the plant-endogenous MIR2911, and whether this potential complexation within the plant matrix could lend further digestive stability and bioavailability to this sRNA.

A large portion of nutrient uptake happens in the intestinal tract, where the epithelial surface is constantly exposed to dietary antigens^40^. The regulation of this intestinal barrier is crucial to control intestinal permeability. Here we used pharmacological (anti-CD3 antibody, aspirin) and genetic (IL-10 mutation) means to increase permeability and potentially impact the uptake of dietary sRNAs. As measured by FITC-dextran, each of these methods improved gut permeability and improved the uptake of the digestively most stable MIR2911 variant without causing a measurable impact on the uptake of less digestively stable sRNAs. It is noteworthy that in anti-CD3-antibody-treated mice, the stable MIR2911 variant (SCR4) reached around 250 fM in the sera (or 1.1 × 10^8^ copies per cell) (Figs 6 and 7). This level approximates endogenous miRNAs such as miR-146a (data not shown). We also attempted other ways to alter the permeability to impact dietary sRNA uptake. Mouse models of hemolytic uremic syndrome (HUS)^41,42^ and chronic and binge ethanol consumption^43^ failed to reproducibly cause changes in intestinal permeability without also producing severe changes in other organ functions (data not shown). Engineering delivery of dietary RNAs may include altering the permeability of the gut, but the work presented here suggests that improved permeability alone is insufficient to drive measurable uptake of most plant-based sRNAs. Again, a reoccurring theme emerges where the majority of dietary RNAs appear too sensitive to digestion to be bioavailable even when gut permeability is improved (Fig. 6D). Recent studies curating available deep sequencing datasets from human tissue and fluids have concluded that previously identified XenomiRs are likely artifacts or misidentified endogenous sequences^44,45^. While we agree with these conclusions-and our data supports the notion that plant dietary miRNAs are probably not bioavailable, we emphasize that MIR2911 is a plant diet-derived bioavailable atypical sRNA with unusual digestive stability^9–11,46,47^. We have consistently measured low background levels of MIR2911 using qRT-PCR in mice and higher levels post-feeding of plant-based diets. Additionally, we have demonstrated that MIR2911 is derived from the 26S rRNA and propagates to ultra-high abundance to aid its bioavailability to consuming mice^46^.

Unrefined or minimally refined plants appear to be core constituents of a “healthy diet”^48^. If dietary plant-based miRNAs are important constituents in these diets their bioavailability is, at best, low. Could these diets be rich in sRNAs that are currently considered degradation products? Mouse feeding studies using a variety of herbs and vegetables demonstrate that the plant 26S rRNA fragment MIR2911 is bioavailable; however, the synthetic form of this sRNA is not readily bioavailable when it is gavage fed to the mice^11^. Our previous work suggests that the rRNAs from the plants is degraded during chewing and digestion, causing massive propagation of MIR2911^21^. The unique biogenesis of MIR2911 may act as a sustained release mechanism during digestion. This degradation/genesis approach maybe a way to thwart complete digestion of dietary sRNAs and allow a small percentage to get into circulation.

Sequencing of mammalian serum sRNAs shows that endogenous 5′ tRNA halves (about 30–40 nt long) are in circulation^49^. Extracellular tRNA halves are present in other biological fluids, such as human semen^50^, media surrounding cell lines^51^, and in plant phloem sap^52^. In the case of tRNA-derived RNA fragments (tRFs), they seem to be involved in translation regulation and gene silencing^53^. tRFs may compete with miRNAs/siRNAs for RISC loading^54^ and may be involved in gene silencing^55^. tRFs can also use unusual regulatory strategies such as protein sequestration^56^ or buffering of other regulatory RNAs^57^. Once considered as “junk” RNAs, tRFs and tRNA halves appear to be important functional elements of eukaryotic and prokaryotic cells^53^. Although most identified tRNA and rRNA fragments still lack known functions, emerging studies suggest that they can co-opt well-characterized regulatory RNA functions (gene silencing, protein sequestration and sRNA buffering). On the other hand, favorable to their potential dietary functions, in plant foods, tRNA fragments also possess the characteristics of degradation-based genesis during digestion of tissues similar to rRNA fragments. Future work with bioavailable sRNAs like MIR2911 will need to be performed to determine if they have bioactive properties within consumers.

## Methods

### Animal studies

The experimental protocol involving mice was approved by the Institutional Animal Care and Use Committee of Baylor College of Medicine. Specifically, the institutional animal protocols AN-2624, AN-6438 and AN-6454 cover the experiments performed in this study. All experiments were performed in accordance with the relevant guidelines and regulations. ICR mice were obtained from the Center for Comparative Medicine at Baylor College of Medicine. Male ICR mice at 7- to 8-week-old were used in most feeding studies. The Interleukin (IL)-10 knockout mice^58^ and the C57BL/6 background mice were kindly gifted by Dr. Gretchen E Diehl at Baylor College of Medicine and were bred and propagated in-house. 8 to 10-week-old male mice were used in the treatment and feeding studies. All experiments were replicated at least three times.

### Drug administration

Mouse monoclonal antibody anti-CD3 (145–2C11) or the purified Armenian Hamster IgG isotype control antibody (Thermofisher Scientific, Waltham, MA) was diluted in sterile 0.9% saline at a concentration of 1 µg/mL. Mice were given a single intraperitoneal injection of either antibodies (15 mg/kg body weight). Aspirin (Sigma, St. Louis, MO) was prepared into a resuspension in 0.5% food grade guar gum. Specifically, aspirin powder and guar gum were weighed and ground in mortar to fine powder and then resuspended in 0.9% saline. The resultant resuspension was administered by oral gavage at 200 mg/kg body weight daily.

### Preparation of synthetic sRNAs

Synthetic sRNAs were obtained from Integrated DNA Technologies (Coralville, IA) with 5′-phosphorylation and 2-O-methylation at the 3′ end nucleotide, to mimic the chemistry of plant-derived miRNAs. The sequence of the miRNAs were as follows: MIR2911 5′-GGC CGG GGG ACG GGC UGG GA-3′; MIR168a 5′-UCG CUU GGU GCA GAU CGG GAC-3′; C7 5′-GGA UCA UCU CAA GUC UUA CGU-3′; 2911m1 5′-AAC CGG GGG ACG GGC UGG GA-3′; 2911m2 5′-GGC CGG GGG ACG GGC UGA AA-3′; SCR4 5′- GGC GGC GGG UCC GGG AGG AG-3′; SCR4–1 5′-GGC GGC GUG GCC GGG AGG AG-3′; SCR4–4 5′-GGC CGG AGG GCG GGC GGU GA-3′; SCR4–5 5′-GGC GGC GGG UCC AGG GGG AG-3′; C7 (an artificial miRNA) 5′-GGA UCA UCU CAA GUC UUA CGU-3′; amiR-RICE^47^ (an artificial miRNA) 5′-UUU GGA AGC AAA GAA GCG GUG-3′. For gavage feeding, miRNAs were diluted in RNase-free phosphate buffered saline (PBS), and each animal was fed 400 pmoles of each miRNA in 300 µL volume^11^. To test the serum stability of the synthetic sRNAs, a cocktail of different sRNAs were directly injected into the mouse tail vein and sera were collected from the mice at 5 min, 30 min, and 60 min post injection. The concentration of the intravenous dose is based on the concentrations of abundant circulatory miRNAs such as mmu-miR-16.

### Serum collection and RNA extraction

Blood was collected via cardiac puncture as previously described^11^. Sera were separated at room temperature followed by centrifugation to remove all blood cells and debris. Total RNA was extracted from 100 µL of sera or other fluids using the miRNEASY Kit (Qiagen, Valencia, CA) following manufacturer’s recommendations. A recent publication suggests that spin columns for purifying small RNAs may be contaminated with microbial RNAs, thereby causing assay artifacts^59^. Negative controls (for example, serum samples that come from mice that are control treated vs antiCD3 treated) suggested that this column issue has little effects in these studies or in the interpretation of the results. A water control which was not subjected to RNA extraction was also used for the qRT-PCR assays.

### FITC-dextran permeability assay, measurement of TNF-α in serum

Intestinal permeability was assessed by oral administration of FITC-dextran 4000 (Sigma, St. Louis, MO). At the beginning of the experiment, food and water were withdrawn for 4 h, and mice were subsequently gavage fed with FITC-dextran solution at 60 mg/100 g body weight. Serum was collected 4 h post-gavage feeding, and FITC-dextran measurements were performed in duplicate by fluorometry (excitation, 490 nm; emission, 530 nm; Cytofluor 2300, Millipore). Serial dilutions of FITC-dextran in PBS were used to calculate a standard curve. Serum TNF-a levels were determined by ELISA kits (Biolegend, San Diego, CA) following manufacturer’s instructions.

### Analysis of sRNA levels by qRT-PCR

Taqman miRNA Assays for let-7dgi^60^, C7, mmu-miR-16 (miR-16), MIR2911, MIR2911m1, MIR2911m2, SCR4, SCR4–1, SCR4–4, SCR4–5, MIR168a and amiR-RICE were obtained from Life Technologies (Carlsbad, CA). Total RNA isolated was used in each reverse transcription (RT) reactions, as previously described^11^. To quantify sRNA/miRNA levels in serum, Let-7dgi was used an endogenous control. To quantify miRNAs in fluids where endogenous controls are not available, 2.5 fmols of synthetic C7 was spiked into the Qiazol lysate during RNA extraction as an exogenous RNA control. qRT-PCR was performed using a Biorad CFX96 Real-Time PCR Detection System, and data were analyzed using Biorad CFX software. Delta-Delta-Ct method was used to calculate relative levels of miRNAs. Absolute concentrations of miRNAs were calculated based on standard curves obtained from serial dilutions of synthetic miRNAs. Given the importance of Ct values in understanding this type of data, the Ct values for SCR4 in sera in non-treated healthy mice was typically 26–27, while the no-template control (NTC) was above 33. In anti-CD3 treated mice (1d after treatment), we consistently measured SCR4 in the sera having Ct values in the range of 22–23. The plant derived MIR2911 could have modifications in sequence length, primary sequence or base modifications. However, we have cloned and sequenced MIR2911 from sera to verify that the consensus MIR2911 sequence is the predominant form found in sera^9^.

### *In vitro* digestion of sRNAs with porcine enzymes

*In vitro* digestion conditions were as previously described. Briefly, the gastric phase was composed of a gastric electrolyte solution (7.8 mM K^+^, 72.2 mM Na^+^, 70.2 mM Cl^−^, 0.9 H_2_PO_4_^−^, 25.5 mM HCO_3_^−^, 0.1 mM Mg^2+^, 1.0 mM NH_4_^+^, 0.15 mM Ca^2+^) with pH adjusted by 1N HCl to 2.0, and porcine pepsin (80 mg/mL) (Sigma, St. Louis, MO); the intestinal phase was formed by adding to the gastric phase an intestinal electrolyte solution (7.8 mM K^+^, 72.2 mM Na^+^, 124.4 mM Cl^−^, 55.5 H_2_PO_4_^−^, 85 mM HCO_3_^−^, 0.33 mM Mg^2+^, 0.6 mM Ca^2+^), 24 mg/mL of bile extract (Sigma, St. Louis, MO) and 40 mg/mL of porcine pancreatin (Sigma, St. Louis, MO), and 1 N NaOH to adjust the pH to 7.0. 1 mL PBS solution containing 10 pmoles each of the synthetic sRNAs were first mixed with 1 mL gastric phase and digested with slow rotation at 37 °C for 60 min. The digestion mixture was then mixed with intestinal phase and digested with slow rotation at 37 °C for an additional 75 min. 100 µL of samples were removed at 30 min, 60 min of the gastric phase, and 5 min, 30 min and 75 min of the intestinal phase for analysis of the levels of surviving miRNAs. 100 µL of pre-digestion samples were used as controls for calculating the percentage of surviving miRNAs.

### *In vivo* digestion of sRNAs in small intestines

To assay miRNAs/sRNA levels in the intestines *in vivo*, the small intestines were harvested 1 hour after gavage feeding of synthetic sRNAs. The levels of miRNAs from the small intestine were determined using the intestinal contents collected by flushing the excised small intestines with 1 mL of PBS solution. 100 µL of the homogenized intestinal contents were analyzed by qRT-PCR for the levels of miRNAs.

### Preparation of cabbage extract and cabbage/chow diet

Fresh cabbage was purchased from a local market. Plant material was first mixed with ice-cold water at 1 g/mL and blended in a mixer. The slurry was sequentially centrifuged at 1000 × g for 10 min, 3000 × g for 20 min, and 10,000 × g for 20 min to remove large particles. The supernatant was then centrifuged at 150,000 × g for 90 min. Supernatant from the ultracentrifuged sample was noted as the cabbage extract. Cabbage/chow diets were prepared as previously described^10^. Briefly, fresh cabbage leaves were cut into shreds and were freeze-dried to 30% of the fresh weight before they were finely ground and used to prepare diets. The cabbage-chow diets were prepared by mixing finely ground chow, plant material, and water at 2:1:2 weight ratios.

### Proteinase K treatment

Mouse sera were mixed with proteinase K (New England Biolabs, Ipswich, MA) at a final concentration of 1 mg/mL, or with the control PBS buffer and were incubated at 55 °C. 100 µL of aliquot was removed for qRT-PCR analysis at 0 min, 5 min, 30 min and 60 min.

### Size-exclusion chromatography

A Sephacryl S-500 column (GE Healthcare, Pittsburgh, PA) was first washed extensively and equilibrated with PBS. 1 mL of pooled mouse sera was loaded and eluted with PBS solution at room temperature^23^. No RNase or protease inhibitors were used during elution. To verify the efficacy of the SEC column, the elution pattern of size standards including thyroglobulin (660 kD), Bovine Serum Albumin (BSA) (66.5 kD) and tyrosine (0.181 kD), and a mouse serum sample were analyzed. The results validated the SEC column to be adequate to separate vesicle and protein fractions^23^. The copies of SCR4 and miR-16 were quantified in each fraction by qRT-PCR and normalized to spiked-in C7. Protein abundance of each fraction was measured by spectral absorbance at 280 nm. Fractions were stored at −20 °C before use.

### Statistical analysis

Statistical analyses were performed with the student t-test formula (two tail, unequal variance) in Microsoft Excel. Significance was set at p < 0.05. Data were presented as means ± SEMs.

## Electronic supplementary material

Datasets
