# Supplementary material for: Intestinal permeability, digestive stability and oral bioavailability of dietary small RNAs
Source: Sci Rep. 2018 Jul 6;8:10253. doi: 10.1038/s41598-018-28207-1 (PMC6035168; doi:10.1038/s41598-018-28207-1)
Supplement: Supplementary file 1 — Datasets [file 41598_2018_28207_MOESM1_ESM.pdf]

Intestinal permeability, digestive stability and oral bioavailability of dietary small RNAs

Jian Yang, Ismail Elbaz-Younes, Cecilia Primo, Danna Murungi, Kendal D Hirschi

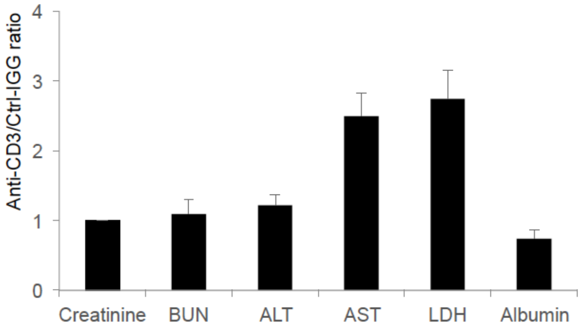

Supplemental Figure S1: Kidney and liver panel of anti-CD3 treated mouse sera.

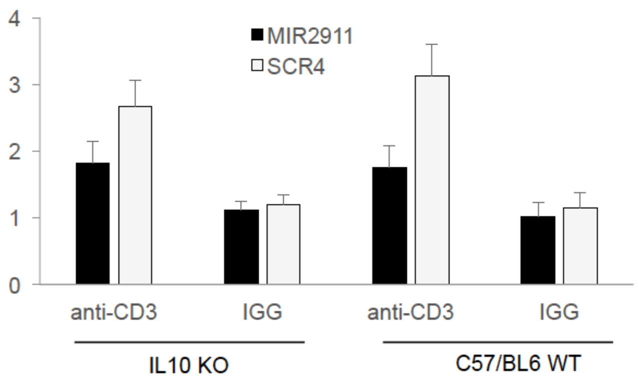

Supplemental Figure S2: IL-10 knockout did not affect uptake of dietary synthetic SCR4 or MIR2911 in mice treated with anti-CD3 for 2days
